# Expression Profiles of *ANGUSTIFOLIA3* and *SHOOT MERISTEMLESS*, Key Genes for Meristematic Activity in a One-Leaf Plant *Monophyllaea glabra*, Revealed by Whole-Mount *In Situ* Hybridization

**DOI:** 10.3389/fpls.2020.01160

**Published:** 2020-08-12

**Authors:** Ayaka Kinoshita, Hiroyuki Koga, Hirokazu Tsukaya

**Affiliations:** ^1^ Graduate School of Science, The University of Tokyo, Tokyo, Japan; ^2^ Exploratory Research Center on Life and Living Systems, National Institutes of Natural Sciences, Okazaki, Japan

**Keywords:** *ANGUSTIFOLIA3*, class I KNOX, Gesneriaceae, indeterminate growth, leaf meristem, *Monophyllaea*, shoot apical meristem, whole-mount *in situ* hybridization

## Abstract

Members of the genus *Monophyllaea* are unique in that they produce no new organ during the vegetative phase in the shoot; instead, one of the cotyledons grows indeterminately. The mechanism of this unique trait is unclear, in part because of the lack of suitable assessment techniques. We therefore established a whole-mount *in situ* hybridization technique, a powerful means of examining spatial patterns in gene expression, for *Monophyllaea glabra*. By using this, we examined the expression pattern of a *SHOOT MERISTEMLESS* (*STM*) ortholog, which is indispensable for the formation and maintenance of the shoot apical meristem (SAM) in typical angiosperms. Expression was confined to the groove meristem (GM), which corresponds to the SAM. We also assessed the expression pattern of *ANGUSTIFOLIA3* (*AN3*), a key promoter for cell division in the leaf meristem. It was expressed not only in the basal meristem (BM) tissue with active cell division in the basal part of the growing cotyledon but also in the GM. The findings suggest that the unusual gene expression pattern of the GM underpins the fuzzy morphogenesis of *Monophyllaea*.

## Introduction

Plants can produce new organs from the meristem throughout their lives, whereas animals complete most morphogenesis during the embryonic stages (Steeves and Sussex, 1989; Graham et al., 2000; Wolpert and Tickle, 2011). The aerial part of typical seed plants, the shoot, is composed of repeating phytomere units (Gray, 1879), each of which consists of a leaf, stem, and axillary bud. All of these components are produced from the indeterminate meristem, the shoot apical meristem (SAM), at the tip of the shoot. The indeterminate nature of the SAM is dependent on the maintenance of stem cells. Because of the indeterminate nature of the SAM, the shoot system is indeterminate. *SHOOT MERISTEMLESS* (*STM*) is indispensable for the formation and maintenance of the indeterminate SAM in model plants; moreover, loss-of-function mutants of this gene lack a SAM (Endrizzi et al., 1996; Long et al., 1996). *STM* encodes a class I KNOX transcription factor. In *Arabidopsis thaliana* four class I KNOX genes, *STM*, *KNAT1*, *KNAT2*, and *KNAT6* support the maintenance of a meristematic state (Hay and Tsiantis, 2010). *STM* and *KNAT1* are expressed only in the SAM (Lincoln et al., 1994; Long and Barton, 1998). *WUSCHEL* (*WUS*) is important for the maintenance of a stem cell niche in the SAM, and loss-of-function mutants lack a SAM (Laux et al., 1996; Mayer et al., 1998; Lenhard et al., 2002).

Leaf primordia are initiated from the flanking region of the SAM and have a determinate meristem, known as the leaf meristem (LM) (Ichihashi and Tsukaya, 2015) that generates leaf lamina cells; therefore, leaves are determinate organs. Genes supporting the LM function have been identified in model plants. *ANGUSTIFOLIA3* (*AN3*)*/GRF-INTERACTING FACTOR1* (*GIF1*) regulates cell division in the leaf meristem by functioning as a transcriptional co-activator of transcription factors, such as *GROWTH-REGULATING FACTOR5* (*GRF5*), in *A*. *thaliana* (Horiguchi et al., 2005; Lee et al., 2009; Kim and Tsukaya, 2015). The leaf of the loss-of-function mutant *an3* has around 30% of the cells of the wild type; the cell number of cotyledons is also decreased (Horiguchi et al., 2005; Lee et al., 2009). In *A*. *thaliana* and *Oryza sativa*, *AN3* is expressed in the basal part of the leaf primordia but not in the SAM (Horiguchi et al., 2011; Shimano et al., 2018). In *Zea mays*, the *AN3* ortholog is expressed from the bottom to the center of the SAM but not at the tip (Zhang et al., 2018).

AN3 protein can move between cells (Kawade et al., 2013; Kawade et al., 2017). Therefore, although the area of *AN3* expression is smaller than that of actively cell dividing area, it matches the meristematic area in leaf primordia, which suggests that it is a determinant thereof. This intercellular movement of AN3 protein is necessary for the proper regulation of leaf meristem activity; in one study, immobilized AN3 protein fused to three GFP molecules did not fully complement the reduced number of leaf cells in an *an3* mutant (Kawade et al., 2013).

One-leaf plants have a developmental system unlike that of typical seed plants such as the model plant, *A*. *thaliana*. They lack a typical shoot system and instead have one indeterminately growing cotyledon and do not produce other new organs, such as stems or foliage leaves, until the reproductive phase (Jong, 1970; Jong and Burtt, 1975; Kinoshita and Tsukaya, 2019). Because they are eudicots, they develop two cotyledons of identical size immediately after germination (isocotyledonous stage), both of which grow. However, after some time, one of the cotyledons (the microcotyledon) stops growing or wither away, whereas the other (the macrocotyledon) continues growing as the sole photosynthetic organ (anisocotyledonous stage), leading to the appearance of harboring a single leaf (Crocker, 1860; Ridley, 1906; Tsukaya, 1997; Nishii et al., 2017). Studies of one-leaf plants have focused on the genera *Monophyllaea* and *Streptocarpus* in Gesneriaceae, which, based on their phylogenetic position, evolved independently. In fact, these genera belong to two different tribes: Epithemateae and Trichosporeae (Jong and Burtt, 1975; Burtt, 1978; Smith, 1996; Smith et al., 1997; Möller et al., 2009; Weber et al., 2013).

Because one-leaf plants have an indeterminate shoot-like character in their macrocotyledon and yet the macrocotyledon is a planar, photosynthetic organ, similar to the leaf of typical plants, the plant body system can be interpreted as “fuzzy morphology” (Rutishauser and Isler, 2001). The term “phyllomorph” has been proposed for this fuzzy morphological unit, which consists of a stem-/petiole-like structure, a petiolode, and an indeterminately growing lamina. One-leaf plants are composed of this single unit (Jong, 1970; Jong and Burtt, 1975; Rutishauser and Sattler, 1985). The growth of the phyllomorph is supported by three meristems: the groove meristem (GM), the basal meristem (BM), and the petiolode meristem (PM). The GM is located at the junction between the macrocotyledon and the petiolode (Jong, 1970; Jong and Burtt, 1975) and is thought to correspond to the SAM because of its position, its tunica-corpus structure (reminiscent of the SAM; Jong and Burtt, 1975; Imaichi et al., 2000; Ayano et al., 2005), and its ability to produce inflorescence (Imaichi et al., 2000; Ayano et al., 2005) although one-leaf plants do not produce new organs in the vegetative phase. The BM is positioned in the basal part of the lamina of the macrocotyledon, which is laterally adjacent to the GM and contributes to lamina growth by active cell division. The BM remains active indeterminately, whereas the LM, which is cell proliferative area in leaf primordia (Ichihashi and Tsukaya, 2015), disappears at a certain developmental stage (Kazama et al., 2010). Because of the indeterminate meristem, the cotyledon in most one-leaf plant species grows for several years; in some *Monophyllaea* species, the inflorescence-bearing mature cotyledon retains the BM (Hilliard and Burtt, 1971; Imaichi et al., 2001). By contrast, the activity of the leaf meristem of *A*. *thaliana* is maintained for only ~1 week (Kazama et al., 2010). The PM is positioned immediately below the GM (Imaichi et al., 2000; Imaichi et al., 2001) or below the two cotyledons (Ayano et al., 2005) and contributes to petiolode growth.

Because the GM does not produce new organs during the vegetative phase, it has been hypothesized that although the GM is corresponding tissue to the SAM, some aspects of SAM functions are lost/suppressed at the GM (Cronk and Möller, 1997; Tsukaya, 1997; Tsukaya, 2000). By contrast, the BM has been hypothesized to have a similar regulatory system as the SAM based on its indeterminate cell division activity (Cronk and Möller, 1997).

To characterize the meristem of one-leaf plants, data on the spatiotemporal expression of the aforementioned key genes are needed. Although genes implicated in the formation and maintenance of meristems in phyllomorphs have been investigated in *Streptocarpus*, no such analyses by *in situ* hybridization have been performed in the genus *Monophyllaea* because of methodological limitations (Ishikawa et al., 2017). Here we established a whole-mount *in situ* hybridization (WMISH) technique for *Monophyllaea glabra* for the first time and investigated the expression of *STM* and *AN3* orthologs to evaluate the GM and the BM of one-leaf plants of the genus *Monophyllaea*.

## Materials and Methods

### Plant Materials and Growth Conditions

Seeds of *Monophyllaea glabra* were originally collected at Srakaew Cave, Thailand (Ishikawa et al., 2017). The strain was maintained by cultivation in growth chambers. Seeds were sown on one third MS medium with 0.8% (w/v) agar, and plants were grown at 22–23°C under white light provided by fluorescent lamps or metal halide lamps under continuous light or short-day (SD; 8 h light and 16 h dark) conditions (Kinoshita and Tsukaya, 2019). The light intensity was ~45 µmol m^−2^ s^−1^.

### Calcofluor Staining and Confocal Microscopy

*M*. *glabra* individuals of 17 DAS fixed and dehydrated for WMISH were rehydrated and immersed in 50% (v/v) calcofluor white stain (Sigma-Aldrich) and 5% (w/v) KOH for ~16 h. The petiolode of *M*. *glabra* was excised, and the samples were mounted with ClearSee solution (Kurihara et al., 2015) and observed with a confocal microscope Fluoview FV10i (Olympus).

### Isolation and Characterization of *CYCB1* and *AN3* Orthologs From *M. glabra*

Total RNA was extracted from inflorescences of *M*. *glabra* with an RNeasy Plant Mini Kit (Qiagen, Germany) following the manufacturer’s protocol. First-strand cDNA was synthesized from total RNA with the SuperScript III First-Strand Synthesis Kit (Invitrogen, USA) according to the manufacturer’s protocol.

Primers for isolating *CYCB1* and *AN3* homologs were designed based on *de novo* assembled sequences obtained from mRNA-seq of *M*. *glabra* (Kinoshita et al., unpublished). The primers for cloning *Mg-AN3* were Mg-AN3_clon-F1 (5′-TTATTACATTACAATCTCGCAGCAC-3′) and Mg-AN3_clon-R2 (5′-AAAAGTGTGACGAAACAAGATCACT-3′). Mg-CYCB1_clon-F1 (5′-CTTCTCAATGGCTTCAAAACAAGT-3′) and Mg-CYCB1_clon-R1 (5′-CAATTAACTGCTAAGTGAAGAAGAAGA-3′) were used to clone *Mg-CYCB1*. The amplicons were ligated to *Eco*RV-digested pZErO-2 plasmids (Thermo Fisher Scientific) and were introduced to *Escherichia coli* to be amplified. We sequenced the plasmids or conducted direct colony sequencing to confirm the sequences of the amplicons. The nucleotide sequences were deposited in DDBJ under accession number LC536022 for *MgAN3-1*, LC536023 for *MgAN3-2*, LC536024 for *Mg-CYCB1-1*, and LC536025 for *Mg-CYCB1-2*.

Since cyclin degradation at a particular cell cycle phase is important for progressing into next cell cycle, the destruction box motif, the key region for the regulation of the cyclin degradation (Glotzer et al., 1991), we referred to Hemerly et al. (1992) to determine the destruction box and used Pfam (https://pfam.xfam.org/; *Cyclin_N*, PF00134; *Cyclin_C*, PF02984) for the other domains to characterize *Mg-CYCB1*. For *Mg-AN3*, Kim and Tsukaya (2015) was referred for the SNH domain.

### Molecular Phylogenetic Analyses

The amino acid sequences other than from *M. glabra* were obtained from the following databases: Phytozome (https://phytozome.jgi.doe.gov/pz/portal.html) for *Amborella trichopoda* (version 1.0), *Oryza sativa* (version 7.0), Zea mays (version 5.0), and *Solanum lycopersicum* (ITAG 2.4), Snapdragon Genome Database (http://bioinfo.sibs.ac.cn/Am) for *Antirrhinum majus* (version 3.0) and TAIR10 (https://www.arabidopsis.org/) for *Arabidopsis thaliana*. GeneBank (https://www.ncbi.nlm.nih.gov/genbank/) (Accession number: AJ250315.1) for CYCB1;1 of *Petunia hybrida*. The amino acid sequences from *M. glabra* were inferred from the longest open reading frames in the cDNA sequences. Amino acid sequences were aligned by MAFFT v. 7.407 in auto mode (Katoh and Standley, 2013), and poorly aligned sequences were trimmed with trimAl v. 1.4. rev. 15 (Capella-Gutiérrez et al., 2009) in automated1 mode. RAxML v. 8.2.12 (Stamatakis, 2014) was used to analyze the phylogenetic relationship with the maximum likelihood method. Bootstrap analysis (Felsenstein, 1985) with 100 replicates was performed with the same software, and phylogenetic trees were generated with FigTree (http://tree.bio.ed.ac.uk/software/figtree/).

### Whole-Mount *In Situ* Hybridization

After confirming the plasmid sequences, we conducted PCR using M13 forward (5′-GTAAAACGACGGCCAGT-3′) and M13 reverse (5′-CAGGAAACAGCTATGAC-3′) primers. The amplicons were used as the template for generating DIG-labelled antisense and sense probes for *Mg-CYCB1-1* and *Mg-AN3-2* by SP6 or T7 polymerase (Roche) using DIG RNA Labeling Mix (Roche). For WMISH, we slightly modified the protocol of Rozier et al. (2014) in order to facilitate the permeabilization of cells. First, we used 4% (w/v) paraformaldehyde (PFA) with 15% (v/v) dimethyl sulfoxide (DMSO) in phosphate-buffered saline with 0.1% (v/v) Tween-20 (PBST) as the fixative unless stated otherwise. Second, the cell wall enzyme treatment was performed for 30 or 60 min with six times diluted stock solution of 1.2% (w/v) Macerozyme R10 (Yakult), 0.5% (w/v) Cellulase Y-C (Kyowa Chemical), and 0.25% (w/v) Pectolyase Y23 (Kyowa Chemical) in PBST. Hybridization was performed at 50°C or 55°C for 3 days. DIG was detected with a DIG Detection Kit (Roche) with a 1:2,000 dilution of the anti-digoxigenin antibody (Roche).

## Results

### Determining the Precise Position of the GM and the BM

**Figure 1:**
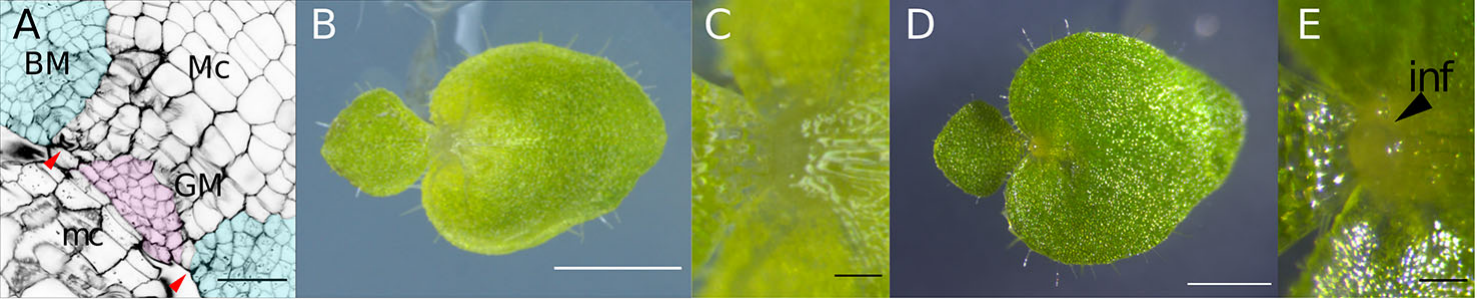
The meristem position in anisocotyledonous-stage *M*. *glabra*. **(A)** Paradermal view confocal micrograph of the tissue structure in an anisocotyledonous individual stained with calcofluor white. The upper right leaf is the macrocotyledon, and the lower left leaf is the microcotyledon. The positions of the GM and the BM are colored pink and pale blue, respectively with an image processing software. The red arrowheads show the position where changes in contour in the macrocotyledon were observed. **(B)** An anisocotyledonous-stage (17 DAS) individual in the vegetative phase grown under continuous light. **(C)** The basal part of the macrocotyledon of the individual in **(B)**. **(D)** An anisocotyledonous-stage (32 DAS) individual in the reproductive phase grown under short-day conditions. **(E)** The basal part of the macrocotyledon of the individual in **(D)**. Black arrowhead, bulging inflorescence produced from the basal part of the macrocotyledon. *BM*, basal meristem; *GM*, groove meristem; *inf*, inflorescence; *mc*, microcotyledon; *Mc*, macrocotyledon. Bar = 50 µm in **(A)**, 1 mm in **(B, D)**, 100 µm in **(C, E)**.

We first defined the GM and the BM anatomically. To determine precisely the position of the GM and the BM from the top of the phyllomorph, we stained the cell walls of anisocotyledonous-stage individuals (17 DAS; **Figures 1B, C** **)** with calcofluor white and take an image of a section including cells of 2nd and 3rd layers in the macrocotyledon with a confocal microscope (**Figure 1A**). Two positionally distinct meristems, the GM and the BM, were evident. One meristem resided in the most proximal part of the macrocotyledon around 100 µm from the mediolateral axis and adjacent to five rows of differentiated cells in the distal part of the meristem. This meristem is the GM—inflorescence was produced at this position, as evidenced by the presence of a bulge (**Figures 1D, E** and **3I**). The other meristem was laterally adjacent to the GM, and its smaller cells were distributed more widely than the GM, both laterally and distally. In the basal part of macrocotyledon, changes in contour were observed at certain points (**Figure 1A**). Hereafter we regard the basal part of the tissue inside these points as the GM and the basal part of the tissue outside it as the BM.

### Isolation and Characterization of Orthologs of *CYCB1*

**Figure 2:**
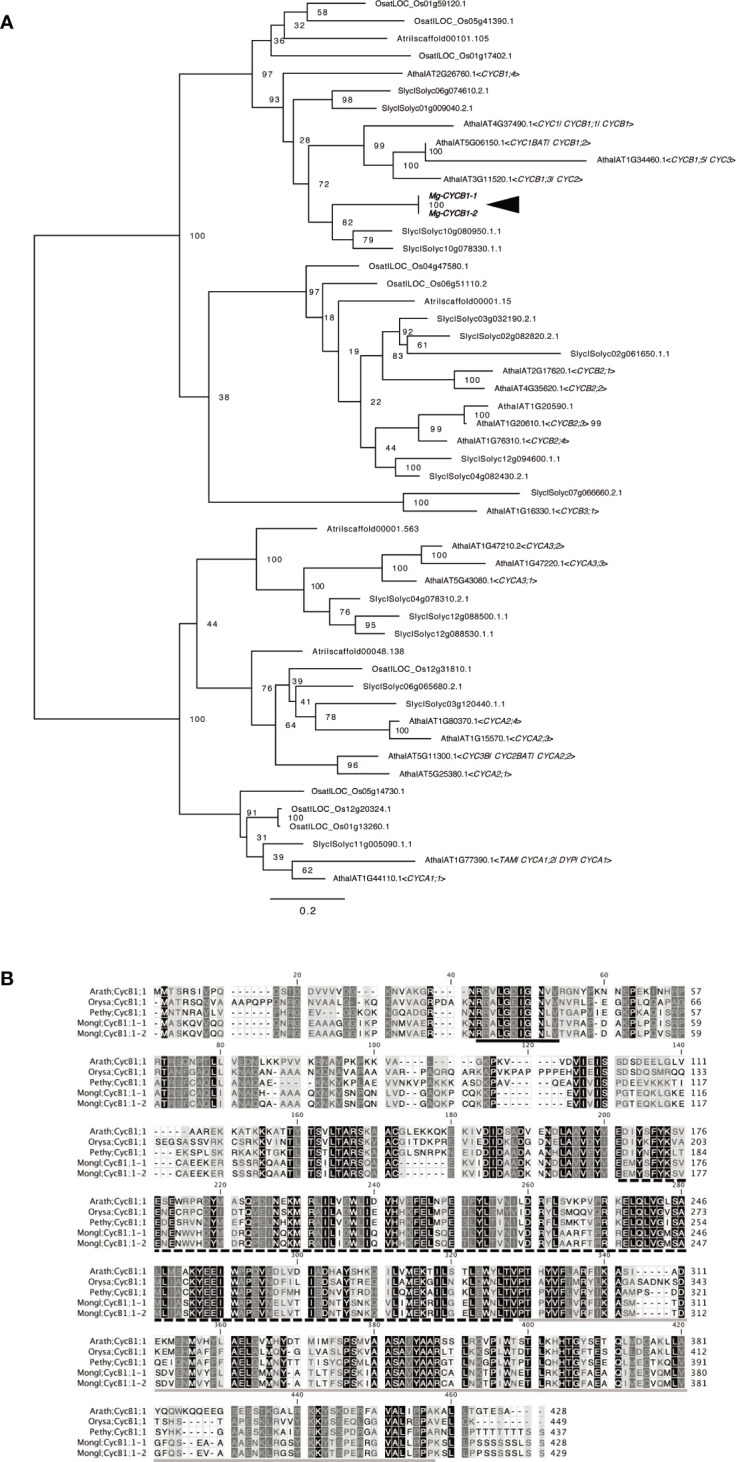
Molecular characterisation of Mg-CYCB1 (Mongl;CycB1;1-1, Mongl;CycB1;1-2). **(A)** Maximum likelihood tree of cyclin amino acid sequences. Bootstrap values are shown at the branches. Amaj, *Antirrhinum majus*; Atha, *Arabidopsis thaliana*; Atri, *Amborella trichopoda*; Osat, *Oryza sativa*; Slyc, *Solanum lycopersicum*; *Zmay*, Zea mays. **(B)** Amino acid sequence alignment of CYCB1 orthologs; dashes indicate gaps. The destruction box, cyclin C-terminal domain, and cyclin N-terminal domain are indicated by black, black hatched, and gray underlining, respectively. The darker background color indicates an amino acid conserved among the five sequences. Arath, *Arabidopsis thaliana*; Orysa, *Orya sativa*; Pethy, *Petunia hybrida*; Mongl, *Monophyllaea glabra*.

*CYCB1*, a marker of the G2/M phase of the cell cycle in *A*. *thaliana* and other model plants, is expressed scattered in tissue with actively dividing cells, such as leaf primordia or floral leaf primordia (Donnelly et al., 1999; Porceddu et al., 1999). We used this gene as the positive control for WMISH of *M*. *glabra*. We isolated cDNA of the *CYCB1* orthologs *Mg-CYCB1-1* and *Mg-CYCB1-2* (**Figure 2A**), the nucleotide and amino acid sequences of which showed 98.3 and 98.8% similarity to each other, respectively. Both harbored a destruction box, a Cyclin_N domain, and a Cyclin_C domain (**Figure 2B**).

### Optimization of WMISH Conditions Based on the *Mg-CYCB1* Expression Pattern

**Figure 3:**
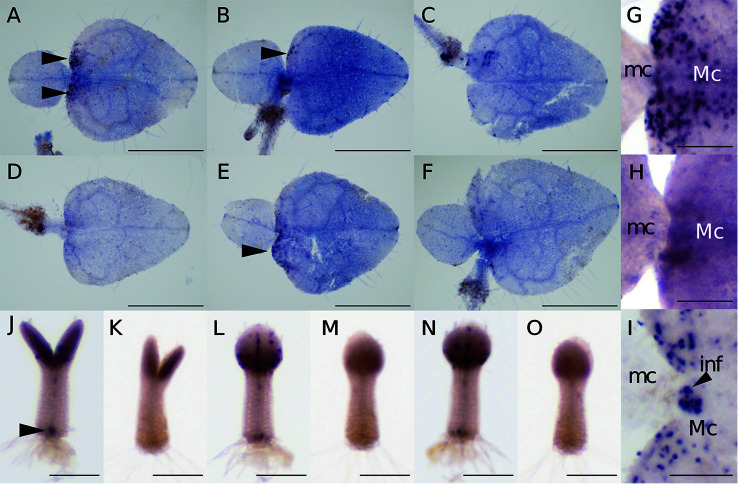
*Mg-CYCB1* expression pattern and meristem positions. **(A–F)** WMISH of *Mg-CYCB1* using an antisense probe with fixation in 4% PFA/15% DMSO **(A, D)**, 4% PFA/1.25% glutaraldehyde **(B**, **E)**, or 4% PFA/1.25% glutaraldehyde/15% DMSO in PBST, respectively. Cell wall enzyme treatment was performed for 1 h **(A–C)** or 3 h **(D–F)**. Black arrowheads indicate signals in the lamina of macrocotyledons. **(G–O)** *Mg-CYCB1* expression pattern at different developmental stages. **(G, H)** Proximal part of the macro- and microcotyledon in an anisocotyledonous individual (17 DAS). **(G)** Antisense probe. **(H)** Sense probe. **(I)** Proximal part of the macro- and microcotyledon at the reproductive stage (38 DAS). **(J–O)** Whole plants at 7 DAS with identical cotyledon size. **(J, L, N)** Antisense probe. The black arrowhead indicates signals in the distal part of the petiolode. **(K, M, O)** Sense probe. **(J, K)** Frontal view. **(L–O)** Side view. *inf*, inflorescence; *mc*, microcotyledon; *Mc*, macrocotyledon. Bar = 1 mm in **(A–F)**, 200 µm in **(G–O)**.

At 15–20 days after sowing, the two cotyledons of *M*. *glabra* were of different sizes. Using individuals at this stage, we optimized the WMISH conditions based on Rozier et al. (2014). Because Tsukaya (1997) showed that BrdU is incorporated into the basal part of the macrocotyledon but not the microcotyledon, we regarded patchy *Mg-CYCB1* signals in the basal part of the macrocotyledon but not in the microcotyledon as a success. It has been known that fixation and permeabilization are key factors for the success in *in situ* hybridization because both factors affect the accessibility of RNA probes and antibodies to target molecules inside cells (Engler et al., 1998; Fuentes and Fernández, 2014). In particular, in terms of the success in the WMISH of plants, permeabilization of cell wall is essential because unlike the classical sectioning *in situ* hybridization, cells are not cut open, so it is more difficult for probes and antibodies to reach the inside cells (Engler et al., 1998; Rozier et al., 2014). Considering above, we tested 4% PFA with 15% DMSO in PBST (**Figures 3A, D** **)**, 4% PFA with 1.25% glutaraldehyde (GA) in PBST (**Figures 3B, E** **)**, and 4% PFA with 1.25% GA and 15% DMSO (**Figures 3C, F** **)** as fixatives with cell wall enzymatic solution treatment (CWES) for 1 h (**Figures 3A–C**) and 3 h (**Figures 3D–F**). DMSO was added to the fixative intending to increase permeabilization (Brodelius and Nilsson, 1983). Fixation with 4% PFA with 15% DMSO in PBST and CWES for 1 h at room temperature was optimal for detecting *Mg-CYCB1* in the basal part of the macrocotyledon (**Figure 3A**) with low background signal. In addition, CWES for 30 and 60 min yielded comparable results, so 30 min CWES was used in subsequent experiments. Whereas anisocotyledonous samples treated with the antisense or sense probe exhibited a pale purple background, patchy dark purple signals were observed only when the antisense probe was used (**Figures 3G, H** **)**. In addition, the *Mg-CYCB1* signal was denser in the BM than in the GM (**Figure 3G**). *Mg-CYCB1* was expressed in both cotyledons at the isocotyledonous stage (**Figures 3J–O** **)**, consistent with the report of Tsukaya (1997) that BrdU is incorporated into both cotyledons immediately after germination. Moreover, the signal was detected in the inner tissue of the tip of the petiolode (**Figures 3J, K**) from which the first root newly emerges as reported in *Monphyllaea singularis* by Imaichi et al. (2001) and in *M. glabra* by Ayano et al. (2005). Therefore, the signals should be from the proliferative cells which start to form the root primordia. We also performed *in situ* hybridization using individuals in the reproductive phase that had started to produce inflorescence meristems (**Figure 3I**). The inflorescence meristem exhibited more signals than the vegetative-phase GM, confirming the suitability of the WMISH condition.

### Expression of *Mg-STM* in *M. glabra*

Because the GM has a tunica-corpus structure similar to the SAM and the inflorescence meristem develops from it, the GM has been hypothesized to be a suppressed SAM that lacks the gene expression necessary for functioning as the SAM (Cronk and Möller, 1997; Tsukaya, 1997; Tsukaya, 2000). To investigate this, Ishikawa et al. (2017) isolated the GM and surrounding tissue by laser microdissection and found that *Mg-STM* expression was higher in the GM than in other tissue. However, they arbitrarily defined the border between the GM and BM meristem, so the spatial pattern of expression of *Mg-STM* was unclear. Therefore, we examined by WMISH the expression pattern of *Mg-STM* at the isocotyledonous and anisocotyledonous stages (7 and 18 DAS, respectively).

**Figure 4:**
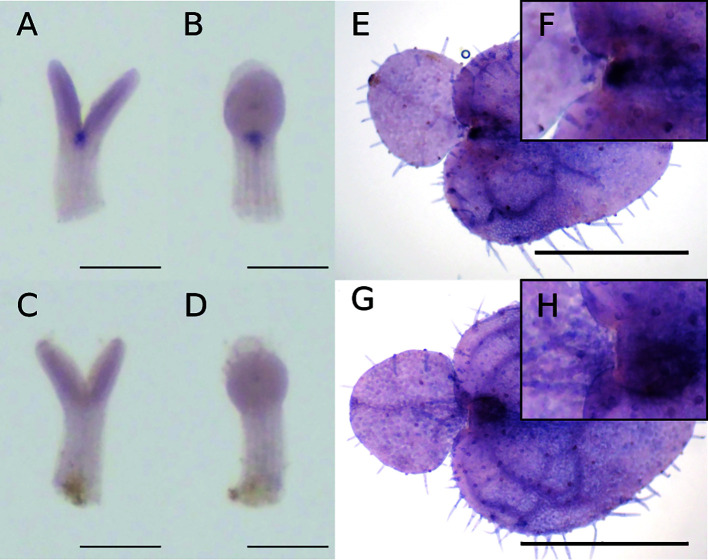
WMISH of *Mg-STM-B* at the isocotyledonous stage (7 DAS) **(A–D)** and anisocotyledonous stage (18 DAS) **(E–H)**. **(A, B, E, F)** Antisense probe. **(C, D, G, H)** Sense probe. **(A, C)** Frontal view. **(B, D)** Side view. **(F)** Magnified image of the GM in **(E)**. **(H)** Magnified image of the GM in **(G)**. Bar = 200 µm in **(A–D)**, 1 mm in **(E, G)**.

At the isocotyledonous stage, *Mg-STM* was expressed between the two cotyledons on the central axis, showing no apparent bias to left or right (**Figures 4A–D** **)**. At the anisocotyledonous stage, *Mg-STM* was expressed only in the most basal part on the midrib of the macrocotyledon, the GM (**Figures 4E–H** **)**.

### Molecular Characterization and Expression Pattern of *Mg-AN3*

**Figure 5:**
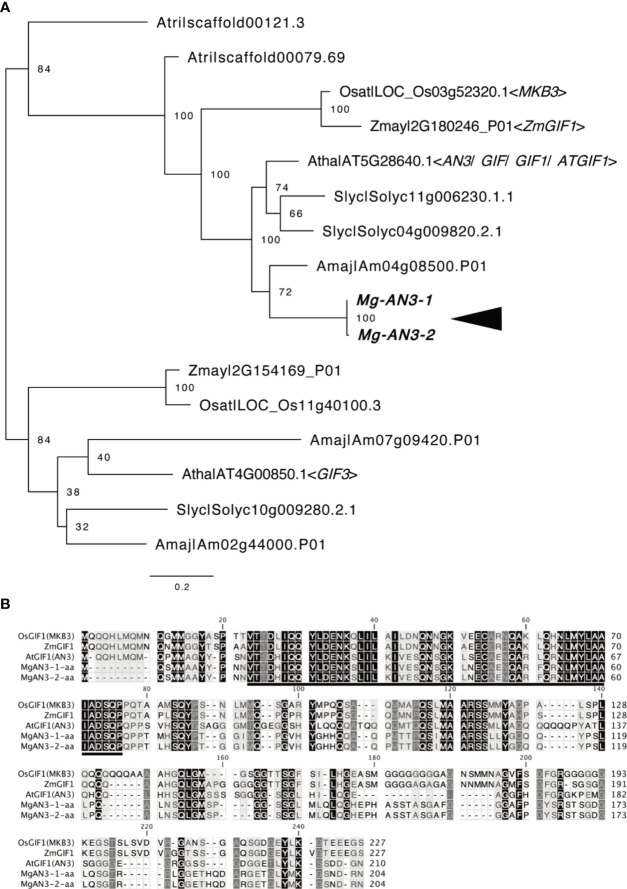
Molecular characterisation of *Mg-AN3*. **(A)** Partial ML phylogenetic tree of the GIF family. Bootstrap values are shown at the branches. Amaj, *Antirrhinum majus*; Atha, *Arabidopsis thaliana*; Atri, *Amborella trichopoda*; Osat, *Oryza sativa*; Slyc, *Solanum lycopersicum*; *Zmay*, Zea mays. **(B)** Amino acid sequence alignment of the AN3 homologs *Mg-AN3-1*, *Mg-AN3-2*, *AtAN3* (*Arabidopsis thaliana* AT5G28640.1), *ZmAN3* (*Zea mays*: Zhang et al., 2018), and *OsAN3* (*Oryza sativa*: Shimano et al., 2018).

Next, we evaluated *AN3* expression in *M*. *glabra* to assess the leaf-meristem-like nature of the BM. We isolated the cDNA of two *AN3* orthologs, *Mg-AN3-1* and *Mg-AN3-2* (**Figure 5A**). The two had 97.4 and 99.5% sequence similarity at the nucleotide and amino acid levels, respectively. The only difference was residue 72, which was methionine or leucine. The putative AN3 protein of *M*. *glabra* possessed an SNH domain (**Figure 5B**), which is conserved among known AN3 orthologs and is necessary for interaction with GRF transcription factors (Kim and Kende, 2004; Horiguchi et al., 2005; Kim and Tsukaya, 2015).

**Figure 6:**
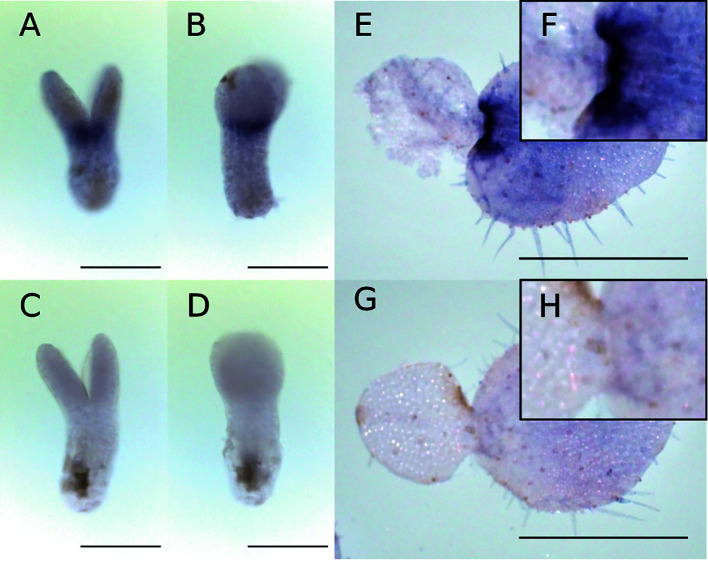
WMISH of *Mg-AN3* at the at the isocotyledonous stage (8 DAS) **(A–D)** and anisocotyledonous stage (17 DAS) **(E–H)**. **(A, B, E, F)** Antisense probe. **(C, D, G, H)** Sense probe. **(A, C)** Frontal view. **(B, D)** Side view. **(F)** Magnified image of the GM in **(E)**. **(H)** Magnified image of the GM in **(G)**. Bar = 200 µm in **(A–D)**, 1 mm in **(E, G)**.

WMISH revealed that *Mg-AN3* was expressed in the basal part of both cotyledons at the isocotyledonous stage (**Figures 6A–D** **)** and in the basal part of the macrocotyledon at the anisocotyledonous stage (**Figures 6E–H** **)** in both the GM and the BM.

## Discussion

### Position of the GM and the BM and Leaf Contour

The leaf contour changed in the proximal part of the macrocotyledon, the position of which was co-terminus with the border between two different kinds of tissue consisting of small cells: one in the proximal centerline of the macrocotyledon and the other laterally adjacent but toward the distal part. Based on this, we defined the former tissue as the GM and the latter tissue as the BM. The change in leaf contour between these meristems might result partly from their different cell division rates. As shown by WMISH of *Mg-CYCB1* at the anisocotyledonous stage, the BM had more signal than the GM, which suggests greater cell division activity. This may explain the heart shape of the leaf (i.e., the protrusion of the lamina toward the proximal part, leaving behind the GM).

### Establishment of a WMISH Technique for *M. glabra*

We established a WMISH technique for *M*. *glabra*, as confirmed by patchy *Mg-CYCB1* expression in mitotic regions in cotyledons, the petiolode, and the inflorescence meristem at the isocotyledonous and anisocotyledonous stages. The technique is rapid because it does not require laborious embedding or sectioning. Moreover, spatial patterns of expression can be easily evaluated because the three-dimensional structure is retained. We could identify gene expression in the GM and/or the BM in a paradermal view, which is difficult with traditional *in situ* hybridization.

WMISH is used less frequently in plant research compared to animal research (Tautz and Pfeifle, 1989; Hemmati-Brivanlou et al., 1990; Herrmann, 1991) and has rarely been used in studies of the photosynthetic organ of plants other than *A*. *thaliana* (Althoff et al., 2014). Our WMISH technique will facilitate further studies of *Monophyllaea* and other non-model organisms.

### *Mg-STM* Expression Pattern

*STM* is essential for the formation and maintenance of the SAM. The loss-of-function mutant *stm* lacks SAM; therefore, no new organ is formed after the cotyledons unfold. This phenotype is similar to that of one-leaf plants (Cronk and Möller, 1997; Tsukaya, 1997; Tsukaya, 2000). *STM* expression and other class I KNOX protein accumulation have been investigated in some phyllomorphs. Harrison et al. (2005) reported that in the one-leaf plant *Streptocarpus dunni*, KNOX I protein was detected in the GM during the reproductive phase but not the vegetative phase, whose result seems to be consistent with the hypothesis that SAM formation/maintenance system is lost/suppressed in the vegetative GM (Cronk and Möller, 1997; Tsukaya, 1997; Tsukaya, 2000). *SdSTM1*, an ortholog of *STM*, is not expressed in aboveground parts of *S*. *dunni* during the vegetative phase. The rosulate species *Streptocarpus* has repeating phyllomorphs (Jong, 1970; Jong and Burtt, 1975) because the GM can produce new phyllomorphs even in the vegetative phase (Nishii and Nagata, 2007). In a rosulate species *S*. *rexii*, *SrSTM1* expression varies according to the stage of the GM and correlates with the production of new phyllomorphs (Mantegazza et al., 2007). Therefore, *STM* expression in the GM is correlated with additional organ formation in *Streptocarpus*.

In this study, *Mg-STM* expression was detected in the proximal part of the future midrib in *M*. *glabra*, consistent with Ishikawa et al. (2017), which suggests that no organ is formed in the GM irrespective of *STM* expression. Moreover, *Mg-STM* was expressed in the area between the two cotyledons at the isocotyledonous stage. This suggests that *Mg-STM* might be necessary for the activity of the GM before macrocotyledon differentiation and is involved in GM formation. Therefore, the molecular mechanism underlying GM formation and maintenance may differ between *Streptocarpus* and *Monophyllaea*. Alternatively, because the class I KNOX gene, *KNAT1*, is expressed in the GM at the no-organ-producing stage in *S*. *rexii* (Nishii et al., 2010), and *KNAT1* is functionally similar to *STM* (Kim et al., 2003), it may replace *STM* in the flat-stage GM of *Streptocarpus*.

Regarding the indeterminacy of the BM, Cronk and Möller (1997) hypothesized that misexpression of SAM-organising genes may cause the indeterminate growth of the cotyledon because these genes maintain the meristematic state. In *S*. *rexii*, *STM* and *KNAT1* orthologs are expressed in the BM. Ishikawa et al. (2017) reported that *Mg-STM* might be expressed in the BM of *M*. *glabra*, causing indeterminate growth. However, our findings suggest that this is unlikely because *Mg-STM* was not expressed in the BM according to our positional definition. Therefore, the mechanisms of phyllomorph growth likely differ between *Streptocarpus* and *Monophyllaea*.

### Prolonged Cell Division Activity in Cotyledons Coincides With *Mg-AN3* Expression

*AN3*, a transcription co-activator expressed in the basal part of leaf primordia, promotes the division of leaf meristem cells in *A*. *thaliana*, *O*. *sativa*, and *Z*. *mays* (Kim and Kende, 2004; Horiguchi et al., 2005; Shimano et al., 2018; Zhang et al., 2018). In isocotyledonous-stage *M*. *glabra*, cell division occurs in both cotyledons, as evidenced by the *Mg-CYCB1* expression pattern. At this stage, *AN3* was expressed in the basal part of each cotyledon. At the anisocotyledonous stage, *Mg-CYCB1* was expressed in the basal part of macrocotyledon but not in the microcotyledon, which indicates that cell division is confined to this area. *Mg-AN3* expression is also confined to the basal part of the area of cell division in the BM, as in leaf primordia of *A*. *thaliana* (Kawade et al., 2017). This suggests that the cell division activity of the BM is, at least in part, supported by Mg-AN3. The greater area of *Mg-CYCB1* than *Mg-AN3* expression could be caused by intercellular diffusion of Mg-AN3 proteins, as in *A*. *thaliana* (Kawade et al., 2017). Therefore, the BM is equivalent to the leaf meristem in terms of the *AN3* and *STM* expression patterns.

### Expression of *Mg-AN3* in the GM

In *A*. *thaliana* and *O*. *sativa*, *AN3* is not expressed in the SAM (Horiguchi et al., 2011; Shimano et al., 2018). In maize, *AN3* is expressed from the bottom to the center of the SAM but not in the tip (Zhang et al., 2018). In this study, an *AN3* ortholog in *M*. *glabra* was expressed not only in the BM but also in the GM, together with an *STM* ortholog. Such complete overlapping of *STM* with *AN3* has not been reported in plants to date. It suggests that the GM has a leaf-meristem-like as well as a SAM-like nature, which may explain the fuzzy plant-body system of one-leaf plants. Moreover, Ishikawa et al. (2017) reported that *Mg-AS1* and *Mg-STM* expression is not mutually exclusive, which suggests that *Mg-STM* and *Mg-AS1* are co-expressed in the BM or GM of *M*. *glabra*. In the present study, we found that *Mg-STM* is not expressed in the BM in WMISH, but Ishikawa et al. (2017) reported that *Mg-STM* was expressed in tissue defined as the BM in their study. This suggests that tissue defined as the BM included a portion of the GM in their study. Nevertheless, *Mg-AS1* expression was detected in the GM suggesting that both *Mg-AS1* and *Mg-STM* are expressed in the GM. The genes that maintain SAM function are believed to repress genes that promote differentiation. For example, in model plants with simple leaves, the SAM-maintaining *STM* suppresses *AS1* (Byrne et al., 2000; Byrne et al., 2002) to maintain an undifferentiated SAM. In addition, the SAM stem cell niche gene *WUS* represses genes that promote differentiation, such as *KANADI1* (Yadav et al., 2013). Thus, the suppression of genes that promote differentiation by genes that maintain the SAM might be impaired in vegetative-stage *Monophyllaea*. This suppression might explain the expression of *Mg-AN3* in the GM. In summary, *Mg-AN3* expression in the GM suggests that it has a leaf-like, as well as a SAM-like, nature.

## Data Availability Statement

The datasets presented in this study can be found in online repositories. The names of the repositories and accession numbers can be found in the main text.

## Author Contributions

AK and HT designed the experiments. AK performed the experiments. AK and HK analyzed the data. AK and HT wrote the manuscript to which HK contributed.

## Funding

This research was supported by a Grant-in-Aid for JSPS Fellows (AK, #19J14140), a Grand-in-Aid for Scientific Research on Innovation Areas (HT, #25113002 and 19H05672) from MEXT and the Graduate Program for Leaders in Life Innovation (GPLLI)/World-leading Innovative Graduate Study Program for Life Science and Technology (WINGS-LST) of the University of Tokyo (AK).

## Conflict of Interest

The authors declare that the research was conducted in the absence of any commercial or financial relationships that could be construed as a potential conflict of interest.
